# The parent-rated short version of the proposed specifiers for conduct disorder (PSCD-SV-P) in Chinese preschoolers: a psychometric evaluation

**DOI:** 10.3389/fpsyg.2026.1775102

**Published:** 2026-02-20

**Authors:** Xiao-Sui Ge, Xing Su, Jie Luo

**Affiliations:** 1 College of Teacher Development, Shaanxi Normal University, Xian, China; 2 Institute of Education, Guizhou Normal University, Guiyang, China; 3 School of Psychology, Guizhou Normal University, Guiyang, China

**Keywords:** Chinese preschoolers, conduct disorder, PSCD-SV-P, psychometric properties, psychopathic traits

## Abstract

**Introduction:**

The proposed specifiers for conduct disorder scale short version (PSCD-SV) is a 13-item shorter version of the original 24-item PSCD developed to assess psychopathic traits in children and adolescents. The current study aimed to examine the factor structure, measurement invariance, and construct validity of the parent-rated PSCD-SV (PSCD-SV-P) in Chinese preschool children.

**Methods:**

A sample of Chinese preschoolers (*N* = 559, *M*_age_ = 4.82, *SD* = 0.86, 46.7% girls) was drawn from two preschools in Guizhou Province through convenience sampling. The PSCD-SV-P and other criterion measures (i.e., CPTI and SDQ) were completed by the preschoolers’ parents.

**Results:**

The CFA results generally supported both proposed first-order four-factor model and four-factor superordinate structure, and the MCFA further verified that the originally proposed first-order factor solution of PSCD-SV-P scores had scalar invariance across children’s genders (i.e., boys and girls). Moreover, the PSCD-SV-P scores had acceptable internal consistency (i.e., McDonald’s *ω*, and MICs), the convergent and criterion validity of the PSCD-SV-P was supported by the expected relations with child psychopathy measures (i.e., CPTI), and psychosocial functioning of children (e.g., conduct problems, hyperactivity, and prosocial behavior).

**Discussion:**

The current study supports the utility of the parent-rated PSCD-SV in assessing psychopathic traits and CD among Chinese preschool children.

## Introduction

Psychopathy, or psychopathic personality, is a multidimensional personality construct encompassing interpersonal (e.g., deceitfulness, grandiosity), affective (e.g., lack of empathy, shallow emotions), and behavioral traits (e.g., impulsivity, irresponsibility), often accompanied by conduct problems (Cleckley, 1976; Hare, 2003; Hare and Neumann, 2008). These traits are closely linked to antisocial personality disorder, and have been consistently associated with aggression, delinquency, and criminal behavior (Cale and Lilienfeld, 2002; Baliousis et al., 2019; DeBlasio and Mojtahedi, 2023). Psychopathy can emerge in early childhood and remain relatively stable over time (van Baardewijk et al., 2008; Colins et al., 2014; Salekin, 2017; Salekin et al., 2022a). Early identification is therefore essential, as it enables timely intervention and may help prevent the development of more severe antisocial outcomes in adolescence and adulthood (Lynam, 2002; Hare and Neumann, 2008; Salekin, 2017). From the developmental perspective, therefore, the selection of the appropriate tools to assess psychopathic traits during childhood is of vital importance.

To date, there are mainly two versions designed to assess psychopathic traits in children, self-report (i.e., child-report) and informant-report (e.g., teacher/parent-report) versions. Given that the manifestations of the psychopathic traits are pervasive and not limited to a single context and/or a certain moment, teachers’ reports of children’s usual and typical behavior at school might provide valuable information, while parents’ reports of children’s behavior at home might be more representative (Colins et al., 2014; Salekin et al., 2018; Ribeiro da Silva et al., 2020; Elhami Athar et al., 2025). Moreover, some studies examined child and parent reports together and indicated that parent-report scores could offer a greater magnitude of effect in predicting some outcomes (Falkenbach et al., 2003; White et al., 2009; Elhami Athar et al., 2025). It is therefore necessary to develop informant-rated measures to assess psychopathic traits during childhood, especially early childhood (e.g., preschoolers).

Several psychometrically-sound instruments have been developed over the course of the past two decades. The Antisocial Process Screening Device (APSD, Frick and Hare, 2001) was designed for children aged 6–13, and relies on parent or teacher ratings to capture a child’s traits such as impulsivity and callousness, but shows weak reliability on the CU subscale (e.g., Colins and Andershed, 2015; Salekin et al., 2018; Ribeiro da Silva et al., 2020). Meanwhile, the Child Psychopathy Scale (CPS, Lynam, 1997) was developed for youth aged 12 or more, and answered by parents of youth to assess psychopathic traits, a one-factor structure (total score only) was usually used (Verschuere et al., 2012). Additionally, the Child Problematic Traits Inventory (CPTI; Colins et al., 2014), developed for children aged 3–12, adopts a three-factor model measuring grandiose–deceitful (GD), callous–unemotional (CU), and impulsive–need for stimulation (INS), and is rated by the child’s teacher or parents. These tools vary in structure, informant, and age range, reflecting active effort to accommodate developmental differences (Wang et al., 2018).

Although these instruments have contributed to the understanding of psychopathic traits during childhood, they lack conduct disorder (CD)/antisocial behavior component, which is particularly noteworthy for researchers and clinicians seeking a more comprehensive exploration of the interplay between psychopathic traits and CD. To address these limitations, Salekin and Hare (2016) developed the Proposed Specifiers for Conduct Disorder (PSCD), which integrates three core psychopathy traits – grandiose–manipulative (GM), callous–unemotional (CU), and daring–impulsive (DI) – with symptoms of CD. The inclusion of CD enhances the instrument’s clinical relevance and predictive power, particularly in identifying high-risk profiles for future behavioral problems (Salekin et al., 2018). Empirical studies have shown that children with high scores across all three PSCD dimensions are more likely to exhibit aggression, rule violation, and criminal recidivism than those with an elevated score on one single trait (Colins et al., 2018; Fanti et al., 2018; Somma et al., 2018; López-Romero et al., 2019a; Colins et al., 2023b; Bellamy et al., 2024; Elhami Athar, 2024; Elhami Athar et al., 2025).

The PSCD has demonstrated promising psychometric properties across a variety of cultural settings, with self-report PSCD versions validated in Chinese adolescents aged 11-17-year-old (Luo et al., 2021), Italian students aged 11–14 years (Muratori et al., 2021), Spanish teenagers aged 12–15 years (López-Romero et al., 2023), Portuguese community (*n* = 648) and forensic youth (*n* = 258) (Ribeiro da Silva et al., 2023), school-attending adolescents aged 14–20 years (Salekin et al., 2024) or forensic (*n* = 227, males) of Belgian youth (Colins et al., 2023a), Iranian school-attending adolescents aged 11-18-year-old (Elhami Athar et al., 2023, 2024) and justice-involved youth aged 9–16 years (Elhami Athar et al., 2025), U.S. children and adolescents (Salekin et al., 2022b; Bellamy et al., 2024). Meanwhile, a parent-rated PSCD version has been initially validated in Spanish preschoolers, providing preliminary support for its applicability to younger populations (López-Romero et al., 2019b). More specifically, López-Romero et al. (2019b) tested and supported the proposed superordinate four-factor structure model (after moving PSCD CU Item 11 to the CD subscale and freeing an error covariance) of the PSCD-parent version (PSCD-PV) in a sample of 2,229 Spanish preschoolers aged 3- to 6-year-old, and this factor model was invariant across children’s gender, as well as the convergent and criteria validity of the PSCD-PV scores was also supported by associations with CPTI scores and by the expected relations with fearlessness, conduct problems (CP), aggression, attention-deficit hyperactivity disorder, oppositional defiant problems, and social competence skill. Then, recent studies have examined and supported the psychometric properties of the parent-report PSCD (PSCD-PV) scores among the U.S. children and adolescents (Bellamy et al., 2024), Iranian school-attending adolescents (Elhami Athar et al., 2024), and Iranian justice-involved youths (Elhami Athar et al., 2025). Furthermore, two empirical studies (Elhami Athar et al., 2024, 2025) have also examined and supported parent–child informant agreement on PSCD scores and psychopathic traits. Specifically, the agreements ranged from 0.49 to 0.68 in school-attending samples, while ranging from 0.65 to 0.86 in the justice involved population.

In addition, a self-report 13-item short version (PSCD-SV; Luo et al., 2021) has also been developed, maintaining the original four-factor structure and demonstrating satisfactory internal consistency, factor validity, and criterion-related validity (Luo et al., 2021; Salekin et al., 2022b; López-Romero et al., 2023). Moreover, a recent study (Bellamy et al., 2023) has preliminarily supported the factor structure, psychometric properties, and criterion-related validity of the shortened (13-item) parent-report PSCD (PSCD-SV-P) scores in a sample of children and youth across the United States. Still, few studies have examined the PSCD-SV within preschool-aged children, and research using parent-report formats in non-Western contexts remains scarce. Cultural differences might influence the expression and understanding of psychopathy (Shou et al., 2021). Indeed, prior studies have showed that certain item(s) within the PSCD measures in Western context might not fully apply to Eastern/Middle East cultures (Luo et al., 2021; Elhami Athar et al., 2023, 2024), to support the proposed factor model of PSCD scores only after removing certain item(s). It is, therefore, of great significance for verifying psychometric properties of parent-report PSCD-SV scores in non-Western cultures (e.g., China).

Although the majority of studies on the PSCD versions (e.g., original and short PSCD versions, self-report and parent-report PSCD versions) have been conducted in Western countries, few studies have tested the PSCD measures in non-Western countries, particularly in Asia, and even the existing research on PSCD measures heavily relies on studies from China (Luo et al., 2021) and Iran (Elhami Athar et al., 2023, 2024, 2025). More specifically, Luo et al. (2021) translated and validated the Chinese self-report version of the original PSCD-24 and preliminary developed the short PSCD-13 in a sample of 1,683 school-attending 11-17-year-old adolescents. These findings supported both a 24-item bifactor model and a 13-item correlated four-factor structure of PSCD scores. Similarly, Elhami Athar et al. (2023) tested and validated the Persian self-report version of the PSCD among 1,506 school-attending 11-18-year-old youth and supported the four-factor hierarchical structure with modified PSCD 19-item scores. Recently, Elhami Athar et al. (2023, 2024, 2025) have further examined and supported the parent–child informant agreement on PSCD scores among Iranian school-attending adolescents (Elhami Athar et al., 2024) and justice-involved youths (Elhami Athar et al., 2025), particularly have promoted the four-factor superordinate model of parent-report PSCD scores in the non-Western population. Indeed, these empirical studies and experiences could help researchers in China use the Chinese version of parent-report PSCD scores to evaluate Chinese samples.

### The current study

The present study aimed to examine the psychometric properties of the parent-rated PSCD-SV (PSCD-SV-P) in a sample of Chinese preschool children. First, based on the originally proposed four-factor model of PSCD-SV scores (Salekin and Hare, 2016; Luo et al., 2021) and informed by prior studies of PSCD-PV scores (e.g., López-Romero et al., 2019b; Elhami Athar et al., 2024, 2025), we used CFA to examine both the first-order four-factor structure and second-order four-factor model of the PSCD-SV-P scores and hypothesized that the two proposed models might fit our data. Then, we also examined the measurement invariance across preschoolers’ gender (i.e., boys and girls) using Multi-group CFA. Following the recent studies (Elhami Athar et al., 2024, 2025), we examined the internal consistency of PSCD-SV-P scores using alpha coefficients, omega values, and means inter-item correlations (MIC). Following the previous studies (López-Romero et al., 2019b), the convergent validity of PSCD-SV-P scores was evaluated with established external measures such as the Child Problematic Traits Inventory (CPTI; Colins et al., 2014), as well as the association between PSCD-SV-P CD scores and CP scores measured using the Strengths and Difficulties Questionnaire (SDQ; Goodman, 1997) also could reflect convergent validity. Finally, the associations between PSCD-SV-P GM, CU, and DI scores and CP, ADHD symptoms, and prosocial behavior indicate criterion validity of PSCD-SV-P. Specifically, it is hypothesized that the PSCD-SV-P scores would be related with CP, ADHD, and prosocial behavior (e.g., Colins et al., 2017; Wang et al., 2018; López-Romero et al., 2019b).

## Materials and methods

### Participants

The data for this study were collected from the children and their parents attending two preschools in Guizhou Province, China. A total of 569 participants were recruited for the study, 10 participants (children aged 7 years) were deleted, the final sample consisted of 559 preschoolers. Children participants ranged in age from 3 to 6 years old, with a mean age of 4.82 years (*SD* = 0.86). Of the parent participants, 72.5% were mothers and 27.5% were fathers; the mean age of the mothers was 35.21 years (*SD* = 4.43, range: 24 to 48 years), and the mean age of the fathers was 36.64 years (*SD* = 4.97, range: 27 to 49 years). Information regarding the preschooler participants’ level at school, number of siblings, family type, and parents’ level of education is presented in Table 1.

**Table 1:** Demographics and statistics for the study sample (*N* = 559).

Variables	Number (%)
Grade level	
Preschool (3–4 years)	159 (28.4%)
Pre-kindergarten (4–5 years)	221 (39.5%)
Kindergarten (5–6 years)	177 (31.7%)
Missing information	2 (0.4%)
Gender	
Girls	261 (46.7%)
Boys	295 (52.8%)
Missing information	3 (0.5%)
Rater	
Mother	405 (72.5%)
Father	154 (27.5%)
Age	
3 years	31 (5.5%)
4 years	174 (31.1%)
5 years	221 (39.5%)
6 years	133 (23.8%)
Only-child status	
Yes	184 (32.9%)
No	373 (66.7%)
Missing information	2 (0.4%)
Family type	
Single parent	13 (2.3%)
Mother and father	535 (95.7%)
Missing information	11 (2.0%)
Parental level of education (mother/father)	
Primary school	3 (0.7%) / 0 (0.0%)
Middle school	18 (4.4%) / 5 (3.2%)
High school	49 (12.1%) / 10 (6.5%)
Bachelor degree or above	333 (82.2%) / 133 (86.4%)
Missing information	2 (0.5%) / 6 (3.9%)

### Procedure

The head of school and the class teachers were informed about the purposes of the current study. Parental information, consent letters, and the questionnaires were sealed in envelopes and sent home with the preschool children. The parents were asked to complete the questionnaires and return them in a sealed envelope to the class teacher within 3 days. This study was reviewed and approved by the Human Subjects Review Committee at the corresponding author’s university (GZNUPSY202111001).

### Measures

#### The proposed specifiers for conduct disorder scale–short version (PSCD-SV)

The PSCD-SV (Luo et al., 2021) is a shorter, 13-item version of the original 24-item PSCD (Salekin and Hare, 2016), designed to assess the four factors of psychopathy: GM (3 items), CU (3 items), DI (3 items), and CD (4 items). Each item is rated on a three-point Likert-type scale (0 = *Not true,* 1 = *Sometimes true*, 2 = *True*). Previous studies have suggested that the psychometric properties of the PSCD-SV are supported in self-report scores (Salekin et al., 2022b; López-Romero et al., 2023).

Following prior studies which have validated parent-rated PSCD versions, such as the Spanish PSCD-parent version (López-Romero et al., 2019b) and the Persian parent-reported PSCD (Elhami Athar et al., 2024), our aim was to design and review a Chinese version of a parent-rated PSCD-SV in accordance with the established Chinese self-report version of PSCD-SV (Luo et al., 2021). As such, the parental participants in the current study were asked to rate the PSCD-SV items based on how well each item described their child. That is, the terms “I and/or me” in the self-report PSCD-SV were changed to “my child” within the parent-report PSCD-SV. Items measured all four factor dimensions of psychopathy, with an example of each being: GM, “Lying is easy for my child”; CU, “My child doesn’t waste time thinking about how he/she may hurt others”; DI, “My child is daring”; and CD “My child has engaged in physical aggression against animals or people”.

#### The child problematic traits inventory (CPTI)

The CPTI (Colins et al., 2014) was originally designed to be a teacher-rated tool to measure children’s psychopathic traits using three factors – grandiose–deceitful (GD; 8 items), callous–unemotional (CU; 10 items), and impulsive–need for stimulation (INS; 10 items) – in early childhood, specifically between 3 and 12 years of age. Each item is rated on a four-point Likert-type scale (1 = *Does not apply at all*, 2 = *Does not apply well*, 3 = *Applies fairly well*, 4 = *Applies very well*). Prior studies have indicated that the psychometric properties of the CPTI are also supported in parent-rated scoring (Somma et al., 2018; Wang et al., 2018; López-Romero et al., 2019a; Luo et al., 2019). The Chinese version of the CPTI has been validated in Chinese children (Wang et al., 2018). In the current study, alpha coefficients for the GD, CU, and INS subscales were 0.79 (MIC = 0.34), 0.88 (MIC = 0.42), and 0.82 (MIC = 0.31), respectively.

#### The strength and difficulties questionnaire (SDQ)

The SDQ (Goodman, 1997) is a 25-item instrument designed to assess the psychosocial functioning of children and adolescents using five subscales: emotional symptoms (ES; 5 items), conduct problems (CP; 5 items), hyperactivity (HY; 5 items), peer relationships (PR; 5 items), and prosocial behavior (PB; 5 items). Each item is rated on a three-point Likert-type scale (0 = *Not true*, 1 = *Sometimes true*, 2 = *Certainly true*). The Chinese version of the SDQ has been validated in Chinese parents (Du et al., 2008). Previous studies have demonstrated significant relationships between psychopathy scores and CP, ADHD, and prosocial behavior (Colins et al., 2017; Wang et al., 2018; López-Romero et al., 2019a), thus three of the SDQ subscales – CP, HY, and PB – were used in the current study. The alpha coefficients for the CP, HY, and PB subscales in the current study were 0.46 (MIC = 0.15), 0.67 (MIC = 0.28), and 0.74 (MIC = 0.36).

### Data analysis

First, descriptive statistics were calculated for the PSCD-SV-P scores using SPSS 25.0. Next, a set of confirmatory factor analyses (CFAs) were conducted to examine and compare the two proposed factor structure models (i.e., original first-order four-factor model, second-order four-factor model) of the PSCD-SV-P using Mplus 7.0. Given that the indices of the skewness and kurtosis for most of the items were beyond −1 or +1, and that the items of the PSCD-SV-P were rated using the three-point Likert-type scale, the robust weighted least squares with mean and variance adjustment (WLSMV) was used (Flora and Curran, 2004). The CFA fit values included the comparative fit index (CFI), the Tucker–Lewis index (TLI), and the root mean square error of approximation (RMSEA). According to Hu and Bentler (1999), CFI and TLI scores above 0.95 with RMSEA indices below 0.05 suggest good fit, while CFI and TLI scores larger than 0.90 with RMSEA scores smaller than 0.08 indicate adequate fit.

Next, following previous studies (López-Romero et al., 2019b; Luo et al., 2021), we tested for measurement invariance (MI) across preschoolers’ gender (i.e., boys and girls). Three levels of MI (i.e., configural, metric, and scalar invariance) were examined to determine whether the factor structure, factor loadings, and item intercepts were equal across the two groups. MI was determined to have been achieved when the differences in fit indices between the constrained and unconstrained models were not statistically significant. As chi-square difference tests are sensitive to sample size, we tested the changes in CFI (ΔCFI) and RMSEA (ΔRMSEA) to compare the nested models. According to Cheung and Rensvold (2002), ΔCFI <0.01 suggests a presence of MI, between 0.01 and 0.02 suggests a likely absence of MI, and >0.02 suggests a definite absence of MI. In addition, following recommendations made by Chen (2007), ΔRMSEA ≥0.015 suggests an absence of MI.

The internal consistency of the PSCD-SV-P scores was then examined using Cronbach’s *α*, McDonald’s *ω*, and MIC values. In terms of clinical significance, Cicchetti and Sparrow (1990) have recommended that alphas of 0.70 to 0.79 indicate “fair,” 0.80 to 0.89 indicate “good,” and 0.90 and higher indicate “excellent” significance. McDonald’s ω was also utilized, for which a value over 0.70 is considered acceptable (Hair et al., 2014). However, it is difficult to use these benchmarks for short item-length scales as the short item scale length may influence alpha and omega values. Therefore, the MIC was also used, with Clark and Watson (1995) outlined guidelines for MIC values, suggesting that acceptable correlation coefficients range from 0.15 to 0.50. Following the results of prior studies, then, the alpha coefficients in the current study were expected to be good for the total scores and modest-to-low for the measured subscales due to the low item numbers of the subscales (Cicchetti, 1994; Clark and Watson, 1995; Tavakol and Dennick, 2011).

Finally, the Pearson correlation coefficients were computed between the PSCD-SV-P scores and an alternative psychopathy instrument (i.e., CPTI; Colins et al., 2014), and the SDQ scores (Goodman, 1997). According to Cohen (1988), correlation coefficient (*r*) values of less than 0.30 suggest weak, between 0.30 and 0.50 indicate moderate, and above 0.50 suggest strong.

## Results

### Descriptive statistics of the PSCD-SV-P

The descriptive statistics of the PSCD-SV-P scores, including means, standard deviations, skewness, and kurtosis, are presented in Table 2. The item–total correlation values of each item were above 0.40 (*p*s < 0.01).

**Table 2:** Descriptive statistics of the PSCD-SV-P scores.

Items	*M*	*SD*	*SK*	*KU*	*r*
1. Lying is easy for my child	0.18	0.41	1.95	2.72	0.44^**^
2. My child takes advantage of others	0.09	0.33	3.65	13.70	0.51^**^
3. My child is a natural storyteller	0.59	0.68	0.72	−0.60	0.52^**^
4. My child does not waste time thinking about how he/she may hurt others	0.29	0.51	1.57	1.58	0.53^**^
5. When people are happy or upset my child does not seem to care	0.16	0.40	2.33	4.78	0.45^**^
6. My child likes it when others are afraid of he/she	0.14	0.42	3.15	9.52	0.46^**^
7. My child likes a lot of change or adventure	0.68	0.67	0.48	−0.78	0.59^**^
8. My child gets a thrill out of doing risky things	0.78	0.67	0.28	−0.78	0.60^**^
9. My child feels like he/she need a lot of stimulation	0.21	0.49	2.37	4.84	0.58^**^
10. My child has (deliberated) stolen things	0.05	0.25	5.90	37.08	0.50^**^
11. My child has engaged in physical aggression against animals or people	0.14	0.38	2.87	7.99	0.48^**^
12. My child has (intentionally) destroyed property	0.11	0.35	3.18	10.15	0.54^**^
13. My child breaks a lot of rules	0.14	0.39	2.90	8.15	0.47^**^
Grandiose–manipulative (GM)	0.29	0.34	1.29	2.15	
Callous–uncaring (CU)	0.20	0.32	1.98	4.36	
Daring–impulsive (DI)	0.56	0.49	0.79	0.29	
Conduct disorder (CD)	0.11	0.24	3.13	13.09	
PSCD-SV-P total	0.27	0.24	1.79	6.45	

SK, skewness; KU, kurtosis; *r*, item–total correlation; PSCD-SV-P, parent-rated proposed specifiers for conduct disorder scale-short version. ** *p* < 0.01.

### Factor structure and measurement invariance of the PSCD-SV-P

**Figure 1:**
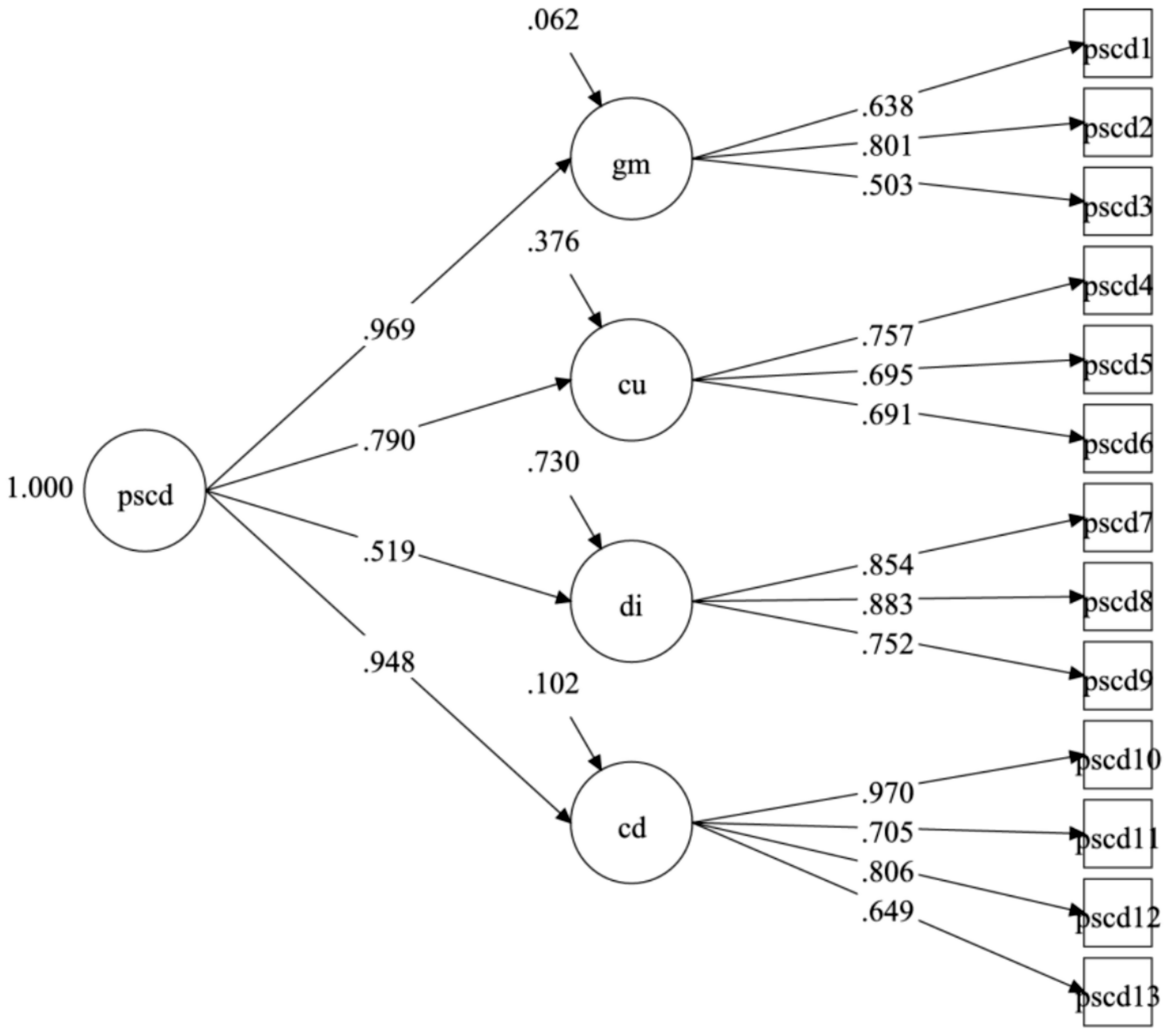
Measurement model for the PSCD-SV-P four-factor superordinate structure. PSCD, proposed specifiers for conduct disorder; GM, grandiose–manipulative; CU, callous–uncaring; DI, daring–impulsive; CD, conduct disorder.

According to the CFA of the PSCD-SV-P scores, the model fit indices of the originally proposed first-order four-factor solution were generally acceptable (WLSMV *χ*^2^ = 196.913, *df* = 59, CFI = 0.918, TLI = 0.891, RMSEA = 0.065), while the second-order four-factor model provided a better model fit (WLSMV *χ*^2^ = 192.461, *df* = 61, CFI = 0.922, TLI = 0.900, RMSEA = 0.062). Overall, these two models of PSCD-SV-P were generally acceptable (Figure 1).

Moreover, the factor loadings for the PSCD-SV-P four-factor model were all acceptable (see Table 3).

**Table 3:** Standardized factor loadings for the PSCD-SV-P four-factor model.

Item	First-order four-factor model				Second-order four-factor model			
	GM	CU	DI	CD	GM	CU	DI	CD
1	0.640^***^				0.638^***^			
2	0.800^***^				0.801^***^			
3	0.502^***^				0.503^***^			
4		0.758^***^				0.757^***^		
5		0.695^***^				0.695^***^		
6		0.690^***^				0.691^***^		
7			0.855^***^				0.854^***^	
8			0.882^***^				0.883^***^	
9			0.752^***^				0.752^***^	
10				0.973^***^				0.970^***^
11				0.704^***^				0.705^***^
12				0.805^***^				0.806^***^
13				0.649^***^				0.649^***^
Factor loadings on the total score					0.969^***^	0.790^***^	0.519^***^	0.948^***^

GM, grandiose–manipulative; CU, callous–uncaring; DI, daring–impulsive; CD, conduct disorder. * *** *p* < 0.001.

Then, the multiple-group confirmatory factor analysis (MCFA) was conducted to examine the MI of the PSCD-SV-P scores across preschoolers’ gender (i.e., boys and girls). More specifically, the MI of the second-order four-factor structure of PSCD-SV-P was not supported, we therefore focused on examining the MI of first-order four-factor model of PSCD-SV-P. First, model fits were tested for the boys and girls separately, and all model fit values were found to be acceptable (boys: CFI = 0.927, TLI = 0.903, RMSEA = 0.066; girls: CFI = 0.958, TLI = 0.944, RMSEA = 0.052). The configural model fit was also acceptable (CFI = 0.941, TLI = 0.922, RMSEA = 0.060). The metric invariance model, in which the factor loadings were set to be equal across boys and girls, was then tested, and found to have an acceptable fit (CFI = 0.941, TLI = 0.927, RMSEA = 0.058), with negligible differences seen in CFI, TLI, and RMSEA values between the configural and metric models (ΔCFI = 0.000, ΔTLI = 0.005, ΔRMSEA = −0.002). These results supported the metric invariance of the PSCD-SV-P scores across preschoolers’ gender. Finally, the scalar invariance model was tested by placing restrictions on all item intercepts to be equal across preschoolers’ gender (i.e., boys and girls). The scalar invariance model fit was satisfactory (CFI = 0.939, TLI = 0.930, RMSEA = 0.057) with negligible changes seen in CFI, TLI, and RMSEA values (ΔCFI = −0.002, ΔTLI = 0.003, ΔRMSEA = −0.001). Thus, the scalar invariance of the PSCD-SV-P scores was also verified across preschoolers’ gender. Furthermore, the independent *t*-tests indicated that no significant differences were found between boys and girls in PSCD four factors, with the exception of the CD subscale scores (*t* = 1.839, *p* = 0.066 for GM; *t* = 0.044, *p* = 0.965 for CU; *t =* 1.051, *p* = 0.294 for DI; *t =* 2.445, *p* = 0.015, *d* = 0.21 for CD).

### Internal consistency of the PSCD-SV-P

In terms of internal consistency values, the Cronbach’s *α*s, McDonald’s *ω*s, and MICs for the PSCD-SV-P total as well as the subscale scores were acceptable, with the exclusion of the αs for GM, CU, and CD. More specifically, the Cronbach’s α for the PSCD-SV-P total score was 0.73 (MIC = 0.18), and although the Cronbach’s αs for the GM (0.38), CU (0.55), and CD (0.47) subscales were lower (excluding *α*_DI_ = 0.72), the MIC values were acceptable for the subscales of GM (0.18), CU (0.29), DI (0.45), and CD (0.19). Moreover, the McDonald’s *ω*s were also acceptable for the PSCD-SV-P subscales of GM (0.69), CU (0.76), DI (0.87), and CD (0.87), and the PSCD-SV-P total score was considered good (*ω* = 0.89).

### Criterion validity of the PSCD-SV-P

To examine the criterion validity of the PSCD-SV-P, we then tested the relationships between the PSCD-SV-P scores and the alternative children psychopathy measures (i.e., CPTI), as well as the associations between the PSCD-SV-P scores with other external variables (i.e., CP, hyperactivity, and prosocial behavior). As Table 4 shows, the PSCD-SV-P scores correlated significantly and positively with the CPTI measures (*r*s = 0.15 to 0.46, *p*s < 0.01). Moreover, the PSCD-SV-P scores were significantly and positively related to children’s CP scores (*r*s = 0.19 to 0.40, *p*s < 0.01), and hyperactivity scores (*r*s = 0.18 to 0.32, *p*s < 0.01), while significant negative correlations were seen with the children’s prosocial behavior scores (*r*s = −0.24 to −0.11, *p*s < 0.01).

**Table 4:** Correlations between the PSCD-SV-P and external measures.

Measure	PSCD-SV-P				
	GM	CU	DI	CD	Total score
PSCD-CU	0.38^**^	1.00			
PSCD-DI	0.27^**^	0.25^**^	1.00		
PSCD-CD	0.39^**^	0.37^**^	0.31^**^	1.00	
PSCD total	0.69^**^	0.66^**^	0.74^**^	0.70^**^	1.00
CPTI-GD	0.41^**^	0.33^**^	0.17^**^	0.32^**^	0.42^**^
CPTI-CU	0.28^**^	0.46^**^	0.15^**^	0.31^**^	0.41^**^
CPTI-INS	0.33^**^	0.36^**^	0.20^**^	0.30^**^	0.41^**^
CPTI-total score	0.38^**^	0.43^**^	0.19^**^	0.35^**^	0.46^**^
SDQ-CP	0.29^**^	0.27^**^	0.19^**^	0.40^**^	0.39^**^
SDQ-Hy	0.22^**^	0.24^**^	0.18^**^	0.26^**^	0.32^**^
SDQ-PB	−0.08	−0.24^**^	0.05	−0.15^**^	−0.11^**^

PSCD-SV-P, parent-rated proposed specifiers for conduct disorder scale-short version; GM, grandiose–manipulative; CU, callous–uncaring; DI, daring–impulsive; CD, conduct disorder; CPTI, Child Problematic Traits Inventory; GD, grandiose–deceitful; CU, callous–unemotional; INS, impulsive–need for stimulation; SDQ, strength and difficulties questionnaire; CP, conduct problems; Hy, hyperactivity; PB, prosocial behavior. ** *p* < 0.01.

## Discussion

This is the first study to examine the psychometric properties of the Chinese parent-report version PSCD-SV (PSCD-SV-P) among Chinese preschool children, focusing on factorial validity, measurement invariance, internal consistency, convergent, and criterion validity. The CFA generally supported the two proposed four-factor models (first- and second-order four-factor structure): GM, CU, DI, and CD dimensions, and the originally proposed first-order four-factor solution was further confirmed to be invariant across gender, as well as had acceptable internal consistency for PSCD-SV-P total and subscale scores especially given the brief number of items per subscales. The correlations between PSCD-SV-P scores and established psychopathy measures in early childhood and external criterion variables supported the convergent, and criteria validity of PSCD-SV-P scores. Overall, the findings preliminary supported the PSCD-SV-P as a valid and reliable tool for assessing psychopathic traits and CD in early childhood in a Chinese cultural context.

### Factor structure of the PSCD-SV-P

The CFA showed that the originally proposed first-order four-factor solution of the PSCD-SV-P demonstrated acceptable model fit in line with prior validations of the PSCD self-report (Luo et al., 2021; Salekin et al., 2022b; López-Romero et al., 2023) and parent-report scores (Bellamy et al., 2024). Importantly, the parent-report PSCD-SV (PSCD-SV-P) is adapted from the original self-report PSCD-SV, and the factor model of PSCD-SV-P might be almost the same as that of the self-report PSCD. The findings suggest that psychopathy is a multifaceted construct that encompasses a set of co-occurring interpersonal, affective, behavioral lifestyle, and antisocial characteristics. In addition, the CFA also supported the proposed second-order four-factor structure of the PSCD-SV-P which is consistent with previous validations of the PSCD parent-report scores (e.g., López-Romero et al., 2019b; Elhami Athar et al., 2024, 2025). The parent-rated PSCD version was initially validated in preschoolers (López-Romero et al., 2019b) and further supported in school-attending adolescents (Elhami Athar et al., 2024) and justice-involved youths (Elhami Athar et al., 2025). The four-factor superordinate model of PSCD-SV-P suggests that psychopathy is not only a multi-dimensional construct encompassing interpersonal, affective, behavioral lifestyle, and CD, but the four different yet interrelated components could be loaded on an overarching latent psychopathy factor (PSCD total) (Salekin, 2017; Salekin et al., 2018). The results of this study extend previous studies by demonstrating that psychopathic traits, as conceptualized by the PSCD framework (Salekin and Hare, 2016), can be reliably measured in Chinese preschool children.

### Measurement invariance of the PSCD-SV-P

Consistent with prior studies (López-Romero et al., 2019b), the current study results also demonstrated strong measurement invariance across preschooler’s gender at the configural, metric, and scalar levels, indicating that the PSCD-SV-P scores express and represent the same information and construct across boys and girls. Additionally, the scalar invariance also suggests that the PSCD-SV-P allows meaningful gender comparisons. More specifically, the tests of factor mean differences indicated that boys have higher PSCD-SV-P CD subscale scores than girls, but effect size was small. These results support that the gender differences in CD, and these differences might show cross-cultural consistency (Muratori et al., 2021). Boys are diagnosed with CD at a higher rate than girls, and boys are more likely to engage in overt aggression, physical fights and rule-breaking behavior. These findings mirror previous evidence of gender invariance for PSCD parent-report scores in children (López-Romero et al., 2019b) and adolescent samples (Elhami Athar et al., 2024) and confirm the suitability of the PSCD-SV-P as a gender-fair assessment tool for early childhood populations.

### Internal consistency of the PSCD-SV-P

In terms of reliability, the PSCD-SV-P total score demonstrated acceptable internal consistency, while subscale reliabilities varied due to the brevity of some subscales. This pattern is consistent with prior studies (López-Romero et al., 2019b; Elhami Athar et al., 2024). Although the Cronbach’s alpha values for the GM and CD subscales were modest (0.38 and 0.47, respectively), the MIC values for all PSCD-SV-P subscales fell within acceptable ranges. In addition, the McDonald’s *ω* for PSCD-SV-P subscales (except for GM subscale) exceeded 0.70, indicating acceptable internal structure and item homogeneity (Hair et al., 2014).

### Convergent and criterion validity of the PSCD-SV-P

With respect to the convergent validity, the PSCD-SV-P subscale scores were significantly and positively related to the corresponding factor scores on the CPTI (Colins et al., 2014; Wang et al., 2018; López-Romero et al., 2019b). Of note, compared to PSCD-SV-P GM, CU, and CD scores, the DI scores correlated weakly with its corresponding CPTI INS scores because the PSCD-SV-P DI subscale might express and reflect daring or sensation seeking rather than impulsivity (Salekin and Hare, 2016; Luo et al., 2021). Moreover, the PSCD-SV-P CD scores demonstrated the strongest positive correlation with CP (e.g., López-Romero et al., 2019b; Elhami Athar et al., 2024, 2025), indicating the PSCD-SV-P CD has satisfactory convergent validity.

In addition, the criterion-related validity was supported by significant associations between the PSCD-SV-P scores and the other external measures. As expected, the PSCD-SV-P subscales scores showed expected associations with the SDQ-measured behavioral indicators. Specifically, the GM, CU, and DI subscale scores were positively related to CP and hyperactivity, while CU and CD subscale scores were negatively associated with prosocial behavior, replicating prior findings in child psychopathy research (Colins et al., 2017; López-Romero et al., 2019b; Muratori et al., 2021). These results suggest that each PSCD-SV-P dimension captures unique and clinically meaningful behavioral correlates (Salekin and Hare, 2016). Interestingly, GM and DI subscales were not significantly associated with prosocial behavior. One explanation for this could be that GM and DI traits might be less directly related to prosocial functioning in early childhood. Future studies are needed to further examine the associations between PSCD-SV-P GM, DI and prosocial behavior among older children and adolescents (Muratori et al., 2021).

### Implications

The results of this study have important theoretical and research implications. First, our findings generally support the two proposed four-factor models of the PSCD-VD-P among Chinese preschoolers. Psychopathy is not only a multifaceted construct encompassing interpersonal, affective, behavioral lifestyle, and antisocial characteristics, as well as these interrelated components could be loaded on an overarching latent psychopathy construct in Western and Chinese populations. Our study also shows that the PSCD-SV-P can serve as a valid and reliable parent-report measurement for assessing psychopathic traits (i.e., GM, CU, DI, and CD) and an overarching psychopathy factor of Chinese school-attending preschoolers, and provides preliminarily support for the scale’s use in cross-cultural research.

### Limitations and future directions

Several limitations of the current study must be noted. First, the sample was drawn from a single region by convenience sampling in Southwest China, which may limit the generalizability of the findings. Future research should use random sampling (e.g., cluster sampling) to obtain more representative participants, and aim to replicate these results across diverse cultural and regional settings. Second, the present study provides preliminary evidence in support of the parent-report PSCD-SV scores, while exclusive use of parent-rated measures may introduce shared method variance, potentially inflating some associations, the multi-informant and multimethod approaches (e.g., teacher-report PSCD-SV) are also needed in future work. Third, although measurement invariance was examined across gender, future studies should further examine measurement invariance across ages, informants and time interval. The PSCD was not originally developed for use with preschool-aged children. Consequently, the developmental appropriateness and content validity of PSCD items for assessing psychopathic traits in early childhood are uncertain. The items would require systematic re-evaluation and potential revision to ensure age-appropriate wording and construct relevance. Finally, future research should employ item response theory (IRT) to examine the functioning of PSCD items in diverse samples, including early childhood populations.

## Conclusion

Despite these limitations, this study expands and confirms the applicable preschool populations which can be assessed using parent-report PSCD-SV, especially in a Chinese context, and verifies the psychometric properties of the PSCD-SV-P among Chinese preschool children. Our findings provide preliminary but compelling evidence in support of the PSCD-SV-P as a valid and reliable tool for assessing psychopathic traits and CP in early screening and research contexts.

## Data Availability

The data supporting the conclusions of this study are available upon request to the corresponding author, JL.
